# 3D Printing Assisted Injection Molding of Chemically Plated W-Cu Composite

**DOI:** 10.3390/ma18081885

**Published:** 2025-04-21

**Authors:** Bo Yuan, Wenwxin Liu, Zhen Wang, Zhongkai Li, Xiaofang Pan, Shurong Xu, Shoujing Mao, Ying Wu, Yangyang Li, Jun Liu

**Affiliations:** 1 School of Materials Science and Engineering, Central South University, Changsha 410083, China; b0716_yuan@163.com (B.Y.); m15211356650@163.com (W.L.); lzk00595@163.com (Z.L.); xfpan00@163.com (X.P.); 213101029@csu.edu.cn (S.X.); mmmsjing@gmail.com (S.M.); yng51_wu@163.com (Y.W.); 18502951561@163.com (Y.L.); 2 School of Materials Science and Engineering, Hunan University of Science and Technology, Xiangtan 411100, China; wangzhen@hnust.edu.cn

**Keywords:** chemical plating, W-Cu composite, photocuring, injection molding

## Abstract

W-Cu composites are widely used in the fields of switch contact materials and electronic packages because of their high hardness, high plasticity, and excellent thermal conductivity, while the traditional W-Cu composite preparation process is often accompanied by problems such as a long production cycle, difficulties in the processing of shaped parts, and difficulties in guaranteeing the uniformity. Therefore, this work developed a chemical plating technique to prepare W-20 wt.% Cu composite powder with a core–shell structure and used this powder as a raw material for powder metallurgy to solve the problem of inhomogeneity in the production of W-Cu composite by the conventional solution infiltration method. Moreover, the work also developed a high-temperature-resistant photosensitive resin, which was used as a raw material to prepare injection molds using photocuring to replace traditional steel molds. Compared to steel molds, which take about a month to prepare, 3D printed plastic molds take only a few hours, greatly reducing the production cycle. At the same time, 3D printing also provides the feasibility of the production of shaped parts. The injection molded blanks were degreased and sintered under different sintering conditions. The results show that the resultant chemically plated W-Cu composite powder has a uniform Cu coating on the surface, and the Cu forms a dense and uniform three-dimensional network in the scanning electron microscope images of each subsequent sintered specimen, while the photocuring-prepared molds were used to prepare the W-Cu shaped parts, which greatly shortened the production cycle. This preparation method enables rapid preparation of tungsten–copper composite-shaped parts with good homogeneity.

## 1. Introduction

W-Cu composites have both high plasticity, the excellent electrical and thermal conductivity of copper, and the high hardness and low expansion coefficient of tungsten [1,2,3,4], which makes them widely used in high-voltage switch contact materials, electronic packages, and electrode materials [5,6].

The great difference in density and melting point between tungsten and copper makes it difficult to produce W-Cu alloys by casting [7,8,9,10,11]. The traditional powder metallurgy process for producing refractory alloys such as W-Cu alloys is mainly prepared by the fusion infiltration method [12,13]. Liu et al. [14] used silver as the active agent and sintered silver and tungsten powders to create a skeleton structure in advance, and then copper powders were uniformly placed on the skeleton, and sintered at 1350 °C to successfully prepare W-Ag-Cu composite, which was improved in density by eliminating the pores of the composite due to the addition of silver. This method can make the W-Cu composite dense, but the production cycle is long, and uniformity is difficult to ensure. In recent years, a series of methods such as mechanical alloying [15,16,17,18], hot isostatic pressing [19,20,21], and plasma sintering [22,23,24] have been developed to prepare more uniform W-Cu alloys. Meng et al. [25] used a planetary ball mill to prepare dense and uniform W-Cu composite coatings with copper powder and tungsten powder as the raw material on a pure copper substrate, and the average thickness of the coatings was 65 μm. These methods can be used to make a W-Cu composite with a higher density and uniformity. To some extent, to solve the problem of uniformity in the W-Cu composite, they are often accompanied by a series of other hindrances, such as high cost, difficulty in preparing parts with complex shapes, and a long production cycle.

In order to solve the above problems of poor uniformity, difficult to process shaped parts, and a long production cycle in the production of W-Cu composite, this work presents a positive and effective process for the preparation of W-Cu composite: (1) Chemical plating on the surface of tungsten powder coated with a layer of homogeneous and dense copper is employed so that in the final sintered product a natural three-dimensional copper network is formed to solve the problems of the traditional W-Cu alloys in the process of preparation of the composition of the segregation of the raw materials; (2) By developing a photosensitive resin that can withstand high temperatures up to 400 °C, the rapid preparation of shaped parts of W-Cu composite is realized by using a light-curing process to prepare injection molds instead of steel molds in the traditional injection molds; (3) Compared with the long production cycle of steel molds in the traditional process (half a month to two months), it takes only a few hours to prepare the injection molds by 3D printing, which greatly shortens the production cycle of W-Cu composite production cycle. The thesis is organized as follows: after the experimental procedure and testing methods are presented in Section 2, the chemical plating process of the composite powder is subsequently described in Section 3, Section 1 and Section 2 shows the effect of sintering temperature on the sintering results, and Section 3 shows the effect of sintering temperature on the sintering results. Therefore, the present work intends to explore, on the basis of previous research, a new production process that can solve the problems of long production cycle, poor uniformity, and difficulty in preparing shaped parts in the existing tungsten-copper composites preparation process. We use chemical plating to prepare W-Cu composite powder and mix the powder with binder to make raw materials. Then, we use light curing to prepare injection blanks for injection molds, and sintering blanks for raw materials in different sintering processes to ultimately achieve the rapid preparation of uniform and consistent W-Cu composite-shaped parts.

## 2. Experimental Method

### 2.1. Materials

Tungsten powder (purity higher than 99%, particle size between 1~10 μm) was purchased from Shanghai Buwei Applied Materials Technology Co., Ltd., Shanghai, China, and copper sulfate was purchased from National Pharmaceutical Group Chemical Reagents Co., Ltd., Shanghai, China; paraffin wax (PW), linear low-density polyethylene (LDPE), stearic acid (SA), ethylene vinyl acetate copolymer (EVA), etc. as binders were also purchased from China Pharmaceutical Group Chemical Reagents Co., Ltd., Shanghai, China.

### 2.2. Experimental

**Figure 1:**
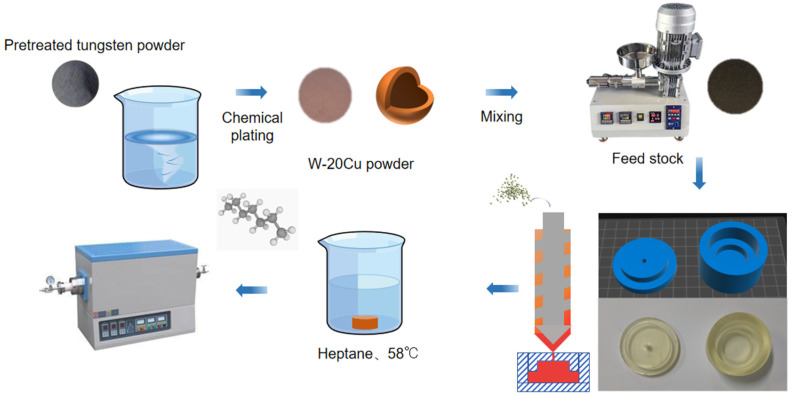
Photocuring-assisted W-20Cu composite injection molding flowchart.

The whole experimental process is shown in Figure 1. Before chemical copper plating, the surface of tungsten powder should be pre-treated. The tungsten powder will be washed with alkali and acid to remove the surface oil and impurities. Then, the activation solution (a mixture of K_4_[Fe(CN)_6_] and AgNO_3_) will make the surface of tungsten powder have a certain catalytic activity. Finally, the activated powder will be put into the copper plating solution. The main components of copper plating baths have evolved over many years, and researchers have formulated them in broadly similar ways. It is the activation bath component that really determines the quality of the plated layer. The copper plating solution used was CuSO_4_ as the copper source, formaldehyde (HCHO) as the reducing agent, disodium ethylenediaminetetraacetate (EDTA) as the complexing agent, NaOH was used to adjust the pH to 12–13, and the Cu content of W-Cu composite powders is controlled by the addition of CuSO_4_ to reach about 20%. The final experimentally determined components for each group were (10 g CuSO_4_-5H_2_O, 5 g NaOH, and 5 mL aqueous HCHO solution). The cleaned powder was placed in a vacuum oven for drying at the end of chemical plating.

The feedstock binder components used in this work include PW and LDPE as the main components, SA and EVA as surfactants and flow agents, and vinyl sulphate (DTD) and p-cresol (BHT) as paraffin antioxidants and metal passivators, and their composition is shown in Table 1. The binder and composite powder were mixed uniformly in a screw extruder at 185 °C. In the final feedstock for injection molding, the volume fraction of chemical W-Cu composite powder was more than 50%, and its mass fraction was more than 94%.

The molds to be used in the injection molding process were 3D printed using the team’s self-developed high-temperature-resistant photosensitive resin and injection molding at 160 °C to prepare blanks with a convex structure. For other shapes of injection molding molds, it is only necessary to create a 3D model and then directly 3D print their molds, compared with the complex process of opening the mold in the traditional injection molding process, the scheme adopted in this work has the advantages of shorter production cycle, lower cost, and so on.

**Figure 2:**
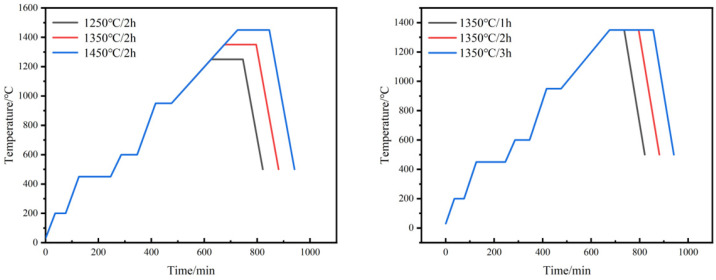
Sintering process of W-20Cu composite.

The obtained green compacts were soaked in n-heptane at 58 °C for 8 h to remove excess paraffin. This step of solvent degreasing is intended to reserve the channel for the subsequent thermal degreasing process, in which the binder is completely pyrolyzed above 450 °C. In order to investigate the influence of different sintering processes on the sintering properties of W-Cu composite, the specimens after solvent degreasing were sintered in a tube furnace protected by an argon atmosphere with different sintering temperatures and sintering times, and the sintering process is shown in Figure 2.

### 2.3. Material Characterizations

After metallographic polishing of the sample to a surface free of scratches, the microstructures of the W-Cu alloy were observed by scanning electron microscopy (CLARA, TESCAN, Ltd., Brno, Czech Republic). Sample analysis was conducted by XRD-7000S diffractometer (Smartlab, Ltd., Tokyo, Japan) using Cu Kα radiation (λ = 1.54060 Å). The density of the sintered body was characterized using Archimedes’ principle. The electrical conductivity test was conducted by an M-6 handheld four-probe tester (Guangzhou Four Probe Technology Co., Ltd., Guangzhou, China). A thermal conductivity test was done with an IFA467HT laser thermal conductivity meter (NETZSCH, Ltd., Selb, Germany).

## 3. Results and Discussion

### 3.1. Phase Composition and Microstructure

**Figure 3:**
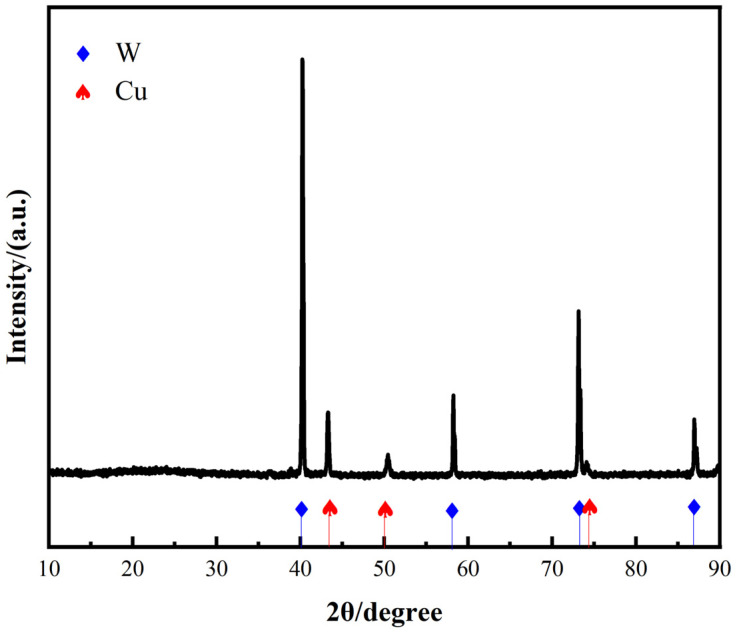
XRD pattern of the W-20Cu composite powder.

The XRD pattern of the W-20Cu composite powder after solution chemical plating is shown in Figure 3, which reveals that the composite powder consists of W and Cu, and that no impurity phases were introduced during the chemical plating of copper.

**Figure 4:**
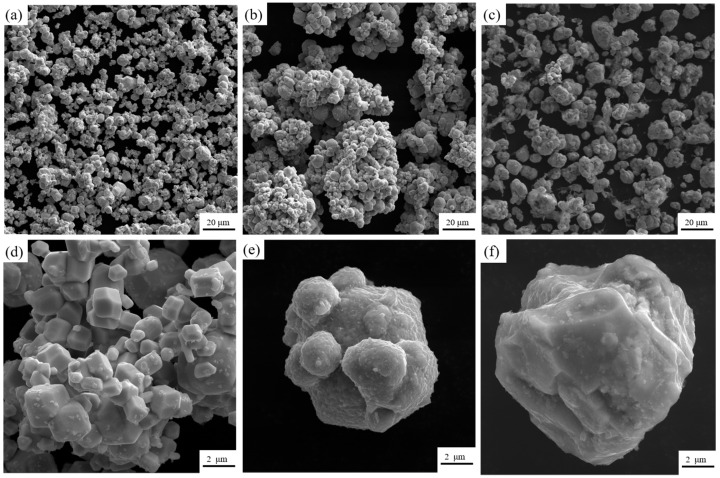
SEM images of different powders. (**a**,**d**) Original tungsten powder; (**b**,**e**) W-20Cu composite powder; (**c**,**f**) Feedstock for injection molding.

Figure 4 shows the scanning electron micrographs of the tungsten powder before pretreatment and the composite powder after chemical plating. From Figure 4a,d, it can be seen that the surface of the tungsten powder particles is clean and tidy, and the particle size is kept in the range of 1–10 μm. It can be seen from Figure 4b,e that the surface of the chemically plated tungsten powder is uniformly coated with a layer of copper with a vesicular structure, and the surface of the copper shows a curved sphere, which suggests that the chemically plated deposited copper may have an obvious selective orientation. Compared with the original W powders, the particle size of the chemically plated composite powders increased and was accompanied by agglomeration, which may be due to the bonding of several dozen composite powders to each other, which is consistent with the findings of many current [26,27,28]. To the naked eye, the composite powder surface showed a bright red color of copper. As shown in Figure 4c,f, from the W-20Cu composite powder feedstock after chemical plating, the middle of the metal composite powder was filled with a large amount of polymer material, which formed a wrapped and encapsulated organizational structure for the composite metal powder particles. During the preparation of the feedstock, the agglomeration phenomenon between the composite powders was improved due to the fact that they were constantly in the densification process, so it can be assumed that the large particle sizes exhibited by the composite powders in Figure 4b,e were due to the presence of a certain thickness of the copper plating layer in contact with each other during the chemical plating process. But, each layer was quickly dispersed during the subsequent mixing process in the single screw extruder.

**Figure 5:**
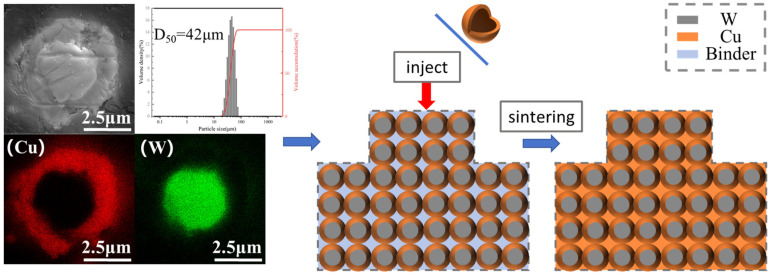
Cross-sectional EDS image of W-Cu composite powder and subsequent schematic diagram of the sintering process.

Figure 5 shows the EDS image of the composite powder. From the image, it can be clearly seen that after chemical plating, where the tungsten powder surface is wrapped in a ring of dense and uniform copper, these surfaces of copper are in the subsequent sintering process to form a natural three-dimensional network and are conducive to the formation of natural and uniform W-Cu composite materials. At the same time, the particle size test shows that the composite powder D_50_ is in about 40 microns, and Figure 4 in the conclusion is mutually corroborated. After chemical plating the composite powder with each other, there are a few or even dozens of powder mutual contact bonds, and in the subsequent mixing process and they were dispersed.

### 3.2. Effect of Sintering Temperature on the Microstructure of Alloys

**Figure 6:**
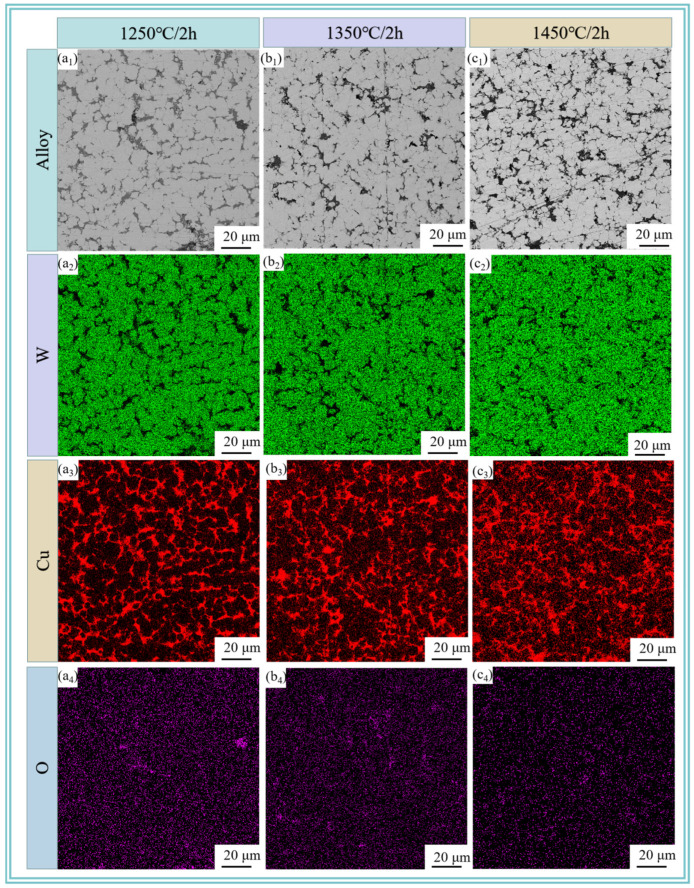
SEM images and elemental distributions of sintered W-20Cu composite: (**a_1_**–**a_4_**) 1250 °C/2 h; (**b_1_**–**b_4_**) 1350 °C/2 h; (**c_1_**–**c_4_**) 1450 °C/2 h.

Figure 6 reveals the metallographic microstructure of W-20Cu composite after sintering the W-20Cu specimens at 1250, 1350, and 1450 °C for 120 min. It can be clearly seen that after sintering, the shape of the edges of the W particles is changed from an irregular polygonal shape to a spherical or sub-spherical shape, and the length of the W particles is limited, which is attributed to the fact that the Cu wrapped around the W particles hinders the growth of W grains. W-Cu alloys are typical ‘pseudo alloys’ [29]. W and Cu are immiscible with each other during the sintering process, specifically, the sintering densification of W-Cu alloys is governed by solid-phase sintering, and the W particles form a skeleton structure during the heating period [30,31]. After the formation of the liquid phase of Cu, the liquid phase and the liquid phase is rearranged through solid-phase sintering, the gaps between the W skeleton are filled with liquid-phase Cu, which makes the composite material more compact. Liquid-phase Cu to fill the gaps between the W skeleton and the liquid phase to further densify the composite. However, as the sintering temperature rises, the W particles do not grow significantly, but some agglomerates appear, which is due to the fact that as the temperature rises, the Cu on the surface of the composite powder undergoes more liquid-phase transformations. Some of the liquid-phase Cu is detached from its original position. It makes the Cu network have a limited limiting effect on the growth of the W particles, and some of the tungsten particles are agglomerated. Figure 5 also shows the results of EDS spectra at different sintering temperatures, and it is observed that a more uniform three-dimensional network of Cu is basically formed in the W-20Cu composite, with good interfacial contact between W-Cu. The composite powder after chemical plating coating contributes to the formation of a three-dimensional Cu network structure. With the increase of sintering temperature, the liquid-phase Cu will not only form a 3D Cu network, but also diffuse to the W-enriched area, which has a positive effect on the improvement of the homogeneity of W-Cu materials.

**Figure 7:**
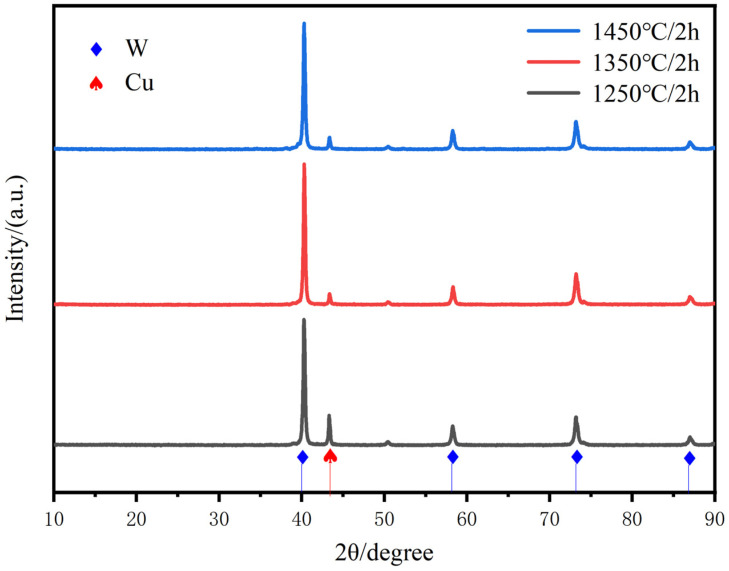
XRD scanning patterns of W-20Cu composite at different sintering temperatures.

Figure 7 gives the enhancement of all diffraction peaks of Cu with increasing sintering temperature, respectively, which may indicate the induced slip of various crystalline surfaces of Cu. The sintering temperature promotes the slip of the copper crystalline surfaces, especially the peaks of the Cu(111) crystalline surfaces are significantly enhanced, clearly indicating the promotion of copper flow and particle rearrangement to achieve homogenization and densification of the alloy matrix. The distribution of the two phases at the cross-section of the plated layer is uniform, and there is no significant difference in the copper content under most of the macroscopic regions. Therefore, the composite powder with chemical plating can be used to prepare a W-20Cu composite with high homogeneity.

### 3.3. Effect of Sintering Time on the Microstructure of Alloys

**Figure 8:**
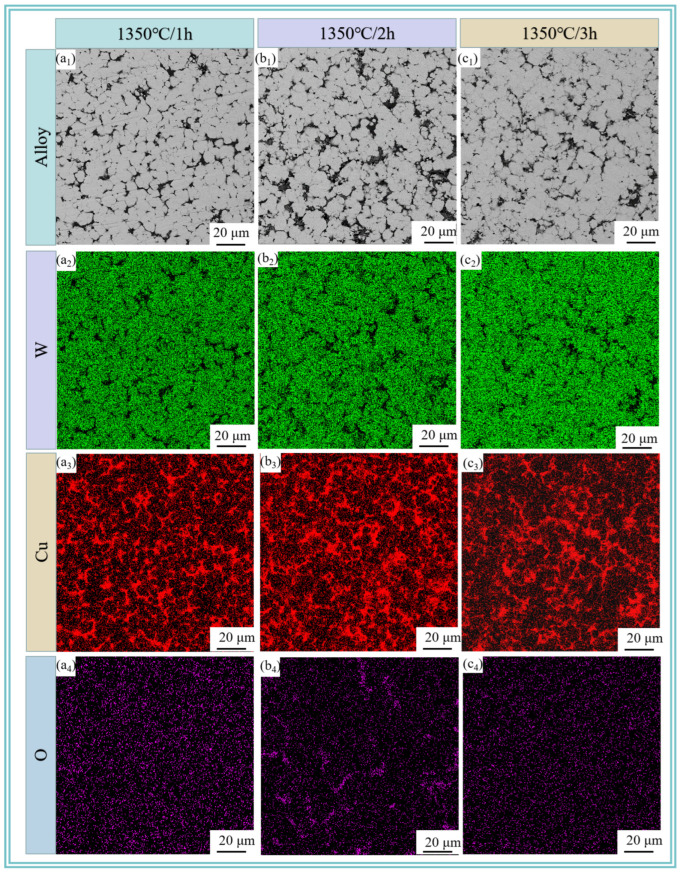
SEM images and elemental distributions of sintered W-20Cu composite: (**a_1_**–**a_4_**) 1350 °C/1 h; (**b_1_**–**b_4_**) 1350 °C/2 h; (**c_1_**–**c_4_**) 1350 °C/3 h.

Figure 8 reveals the microstructure of W-20Cu composite after sintering the W-20Cu specimens at 1350 °C for 60, 120 and 180 min, respectively, where the grey tissue is the skeleton-like tungsten phase, and the light black tissue is the adherent phase copper at its periphery. A small number of tiny pores can be observed in Figure 8b_1_, and the copper phase is distributed in the interstices of tungsten particles. With the increasing sintering time, the pore size distribution first becomes more obvious and then decreases, which is due to the fact that in the process of high-temperature liquid-phase sintering, the wettability between the tungsten and copper is not very good at 1350 °C [8,32,33,34] and the liquid-phase copper will produce evaporation loss after generation. At the same time, due to the incomplete liquid-phase wettability, the interfacial energy of the solid–solid interface is lower than that of the solid–liquid interface, and solid particles come in contact with each other, making the copper melt permeation volume higher, so that the amount of copper melt penetration is reduced, part of the copper had to overflow. Moreover, the sintering temperature is above the melting point of copper, and the copper will inevitably evaporate [35]. The formation of pores between the tungsten particles due to the intervention of external gases and the shrinkage of the metal in the liquid state is greater than that in the solid state, resulting in a large number of shrinkage holes, which ultimately leads to an increase in the porosity of the sintering. As the sintering time increases, the pores have more time to shrink, and the tungsten particles tend to aggregate and grow [36,37,38]. Meanwhile, there is also a process of reverse fusion infiltration in the liquid-phase copper, but due to the volume contraction after sintering, the tungsten shrinks to form closed pores, the copper liquid can not be completely infiltrated, and the prolonged sintering time leads to the gradual spheroidization and shrinkage of the closed pores, but does not disappear [39]. The increase in porosity after 3 h sintering may be due to the large amount of copper loss, and at the same time, the closed pores inside the copper phase and adjacent to the tungsten are exposed with the loss of the copper phase, resulting in an increase in porosity.

**Figure 9:**
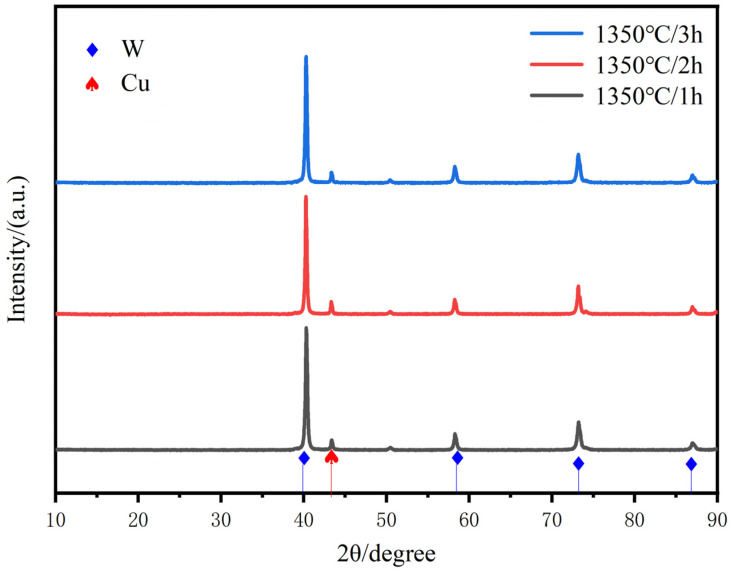
XRD scanning patterns of W-20Cu composite with different sintering times.

Figure 9 shows the XRD diffraction patterns for different times of sintering at 1350 °C. The results show that no new phases are generated with the extension of sintering time, and the position and intensity of the XRD diffraction peaks of W do not change significantly in this process. Compared with the sintering temperature, the increase in sintering time does not cause any significant slippage of one of the crystal surfaces of copper, except for the local growth of W particles.

### 3.4. Influence of Process Parameters on Final Cinder Properties

Table 2 shows the effect of different sintering temperatures and sintering times on the density and properties of the W-20Cu composite. With the increase of sintering temperature, the density and thermal conductivity of the composites increase, and the resistivity decreases, which was attributed to the fact that the increase of temperature promoted the slip of copper and formed a more complete three-dimensional network of copper in the composite system. The electrical and thermal conductivity of metallic materials are both mainly related to the electron movement within the metal, the free electrons within the metal continuously collide with the metal cations to transfer energy and thus heat; similarly, the electrical conductivity of the metal also relies on the directional movement of the free electrons within the metal, and these Cu three-dimensional lattices provide a bridge for the free movement of electrons in the alloy material. Densification, electrical conductivity, and thermal conductivity increase with the formation of the copper 3D network as the sintering time increases, but with further extension of the sintering time, solid-phase sintering occurs in tungsten particles with high activity and a certain amount of closed pores before the copper phase escapes from the interconnected pores. These closed pores could not be eliminated by volume diffusion and surface diffusion during the subsequent solid-phase sintering stage, resulting in less densification of the sintered body.

### 3.5. Discussion and Optimization of Low Densities

The low densification of the composite may be due to the following factors, considering the raw materials.

(1) The matrix powder used in the initial surface chemical plating is irregularly shaped tungsten powder with a scale between 1–10 microns, which is insufficiently driven during sintering. It is considered that the use of spherical tungsten powders with a smaller particle size as the matrix powder may be able to promote the densification of W-20Cu composite during the sintering process.

(2) Excessive binder content in the feed. In the powder metallurgy process, a too high binder volume fraction tends to result in high porosity, and these binders tend to leave pores in situ during subsequent thermal degreasing. But, due to the high specific gravity of W-Cu, it is difficult for a smaller amount of binder to mix uniformly with a large amount of W-20Cu composite powder. Comprehensive problems between the two, consider, on the basis of the existing appropriate reduction of binder content and binder in the proportion of LDPE, reducing the volume fraction of binder in the billet at the same time as much as possible to improve the fluidity of the feedstock, while consider reducing the degreasing temperature rate, so that the binder is slowly and completely removed, to give the W-20Cu composite material sintering densification more space.

(3) When injection molding the blanks, the injection blanks are often not dense enough due to the limited pressure that the photocuring molds can carry, and the molds themselves may have micro-cracks when the pressure is too high. Consider improving the raw material of the mold to increase its load-bearing capacity and to improve the density of the injected blanks.

## 4. Conclusions and Vision for the Future

In this paper, a rapid method for the preparation of highly homogeneous W-20Cu composite with a heterogeneous structure is presented with the following results:

(1) SEM images show that the Cu chemically plated on the surface of W-20Cu composite powder has a continuous three-dimensional network structure with dense coating, the coating is uniform on the surface of W particles, and the composite powder has a light red color, which is capable of realizing the preparation of W-C alloys with high homogeneity by using this powder as a raw material.

(2) A high-temperature resistant mold suitable for injection molding was prepared by photo-curing, and based on this mold, the rapid manufacturing of W-Cu alloy with heterogeneous structure was realized, and it has a significantly shorter production cycle compared to traditional steel molds.

(3) The results show that the rapid preparation of W-Cu composite with high homogeneity was successfully achieved by using light-curing-assisted injection molding of W-Cu composite. The use of 3D-printed molds instead of steel molds can shorten the production cycle while significantly reducing the cost, providing a new solution for the production of the W-Cu composite.

In view of the low density of the composites caused by the lack of maturity of the new process, which may be due to the decomposition of organic residues in the high temperature sintering process and its mechanism of porosity and interfacial strength, the kinetic mechanism of densification and its correlation with the interfacial evolution should be deeply investigated in future research. The influence of sintering time, sintering temperature, original powder particle size, and binder mass fraction on the final density of the composites should be further explored based on the numerical simulation. The effects of sintering time, sintering temperature, original powder particle size, and binder mass fraction on the final densification of composites should be further investigated based on numerical simulations.

## Figures and Tables

**Table 1:** Binder components.

Element	Content/wt%	Decomposing Temperature/°C
LDPE	60	350–450
PW	30	234
EVA	4	230
SA	4	376
BHT	1	265
DTD	1	200–250

**Table 2:** Properties of W-Cu composite under different sintering conditions.

Cementing Process	Density/%	Electrical Resistivity /μΩ·cm	Diffusivity/(mm^2^/s)	Conductivity/ (W/(m·K))
1250 °C/2 h	68.30	22.80	51.64	109.80
1350 °C/2 h	70.06	21.60	50.96	112.68
1450 °C/2 h	74.90	19.84	50.24	114.23
1350 °C/1 h	69.30	19.50	51.64	110.10
1350 °C/2 h	71.20	18.27	49.93	112.49
1350 °C/3 h	69.93	19.30	51.08	111.42

## Data Availability

The original contributions presented in this study are included in the article. Further inquiries can be directed to the corresponding author.

## Abbreviations

The following are the English abbreviations and their full names appearing in the article.

**Abbreviations**	**Full Name**
LDPE	Low Density Polyethylene
PW	Paraffin Wax
EVA	Ethylene Vinyl Acetate
SA	Stearic Acid
BHT	P-cresol
DTD	Vinyl Sulphate
XRD	X-Ray Diffraction
SEM	Search Engine Marketing
